# Case Report: Complete pathological remission of human chorionic gonadotrophin-producing gallbladder carcinoma with multiple liver metastases after treatment with chemotherapy plus an immune checkpoint inhibitor

**DOI:** 10.3389/fimmu.2023.1173520

**Published:** 2023-09-29

**Authors:** Qianwen Wang, Yunchuan Mu, Shunxian Ji, Yang Liu, Yanbo Lou, Shumei Wei, Xin Dong, Bo Zhang

**Affiliations:** ^1^ Department of Surgery, Fourth Affiliated Hospital, School of Medicine, Zhejiang University, Yiwu, China; ^2^ Department of Pathology, Fourth Affiliated Hospital, School of Medicine, Zhejiang University, Yiwu, China; ^3^ Department of Pathology, Second Affiliated Hospital, School of Medicine, Zhejiang University, Hangzhou, China; ^4^ Department of Surgery, Second Affiliated Hospital, School of Medicine, Zhejiang University, Hangzhou, China

**Keywords:** gallbladder carcinoma, human chorionic gonadotrophin, immune checkpoint inhibitor, chemotherapy, pathological complete response

## Abstract

**Background:**

Gallbladder carcinoma (GBC) producing human chorionic gonadotrophin (HCG) is an extremely rare and highly invasive tumor with a poor prognosis. This unfavorable clinical outcome is partly due to the aggressive nature of the tumor and its insensitivity to chemotherapy.

**Case presentation:**

We herein report a case of primary GBC producing HCG with liver metastases in a 58-year-old woman. The patient presented with a markedly elevated β-HCG level and a mass in the gallbladder with multiple liver metastases. A definitive diagnosis was obtained after a needle biopsy of the liver metastases, showing poorly differentiated carcinoma with large-scale necrosis and strong positivity of immunostaining for HCG in tumor cells. The patient received chemotherapy (gemcitabine plus capecitabine) combined with carrellizumab, an immune checkpoint inhibitor (ICI). Pathological complete response was achieved after eight courses of combined therapy, which was confirmed by pathological analysis of resected specimens. After surgery, two courses of chemotherapy plus ICIs were adopted again. Complete response remained for approximately 1 year up to the present. Tumor tissue was collected to perform immunostaining of PD-L1, whole-exome sequencing, and RNA-seq. Low-TMB (1.51 mut/Mb), MSS, and high PD-L1 expression (TPS ≥ 50%) were observed in the tumor. Besides, the dominant types of infiltrating immune cells were macrophage and CD4+ T cells. Compared to other gallbladder adenocarcinoma without HCG, the proportion of M1 macrophage was at a higher level and the gene sets of MYC targets v1 and PI3K/AKT/mTOR signaling were highly expressed in our case. To the best of our knowledge, this is the first case report of complete remission of HCG-producing gallbladder carcinoma with liver metastases after chemotherapy combined with an immune checkpoint inhibitor. Furthermore, this is also the first report that described the tumor genetic feature and tumor immune microenvironment atlas of HCG-producing GBC.

**Conclusion:**

chemotherapy plus an immune checkpoint inhibitor may provide a potentially curative option for gallbladder carcinoma with HCG production.

## Introduction

Human chorionic gonadotropin (HCG) is a chemical secreted by syncytiotrophoblast cells of the placenta, which is commonly observed in pregnancy or uterus cancers (1). Elevated HCG levels can also be helpful in identifying a variety of extragonadal malignancies, including prostate cancer, colorectal cancer, lung cancer, and breast cancer (2–5).

Primary gallbladder carcinoma (GBC) with ectopic HCG is exceedingly rare and is rapidly growing, widely metastasizing, and highly invasive of surrounding tissues. There are only a few cases reported in the literature until now (**Table 1**) (6–13). The prognosis is considerably poor due to the aggressive nature of the tumor and lack of a definitive treatment strategy. Chemotherapy regimens, used successfully for gestational choriocarcinoma, appear not to be as effective for GBC with HCG secretion (8, 14). In addition, some studies also found that surgery combined with Cisplatin- or Gemcitabine-based chemotherapy is effective for this disease, which can improve overall survival (9, 11). Due to the rarity of GBC producing HCG, the optimal treatment has not been well established and further research is needed. Especially in the era of immunotherapy, there is no report on the application and efficiency of immune checkpoint inhibitors (ICIs) on this rare type of GBC, as well as the description of features of the tumor immune microenvironment (TIME).

**Table 1:** Case reports of gallbladder carcinoma producing HCG.

Reference	Age/sex	Symptom	Treatment	Prognosis
Guo KJ et al (6)	68/F 65/F 68/F 68/F 69/M 61/M 66/M 61/F 62/F	NA	Surgery Surgery Chemotherapy Chemotherapy Surgery Surgery Surgery Chemotherapy Chemotherapy	Died (3 months) Died (8 months) Died (NA) Died (NA) Died (3 months) Died (3 months) Living (19 months) Died (NA) Died (NA)
Fukuda T et al. (7)	83/F	Abdominal distension	No treatment	Died (NA)
Abu-Farsakh H et al. (8)	29/F	Intraabdominal bleeding	Surgery+ Chemotherapy	Died (1 months)
Wang JC et al. (9)	48/F	Abdominal pain	Surgery+ Chemotherapy	Died (12 months)
Macdonald MC et al. (10)	41/F	Vaginal bleeding and nausea	Chemotherapy	Died (7 months)
Sato S et al. (11)	79/M	Jaundice	Surgery+ Chemotherapy	>11 months/PFS
Goto A et al. (12)	61/F	Fever	Chemotherapy	NA
Leostic A et al. (13)	31/F	No symptom	Surgery	PD/NA

PD, progressive disease; PFS, progression-free-survival; NA, not accessed.

Herein, we reported a GBC case producing HCG, in which the patient completely responded to chemotherapy plus ICIs, and the tumor tissue was collected to perform immunostaining of programmed death-ligand 1 (PD-L1) and whole-exome sequencing (WES) to explore the genetic features and TIME of GBC producing HCG.

## Case presentation

**Figure 1:**
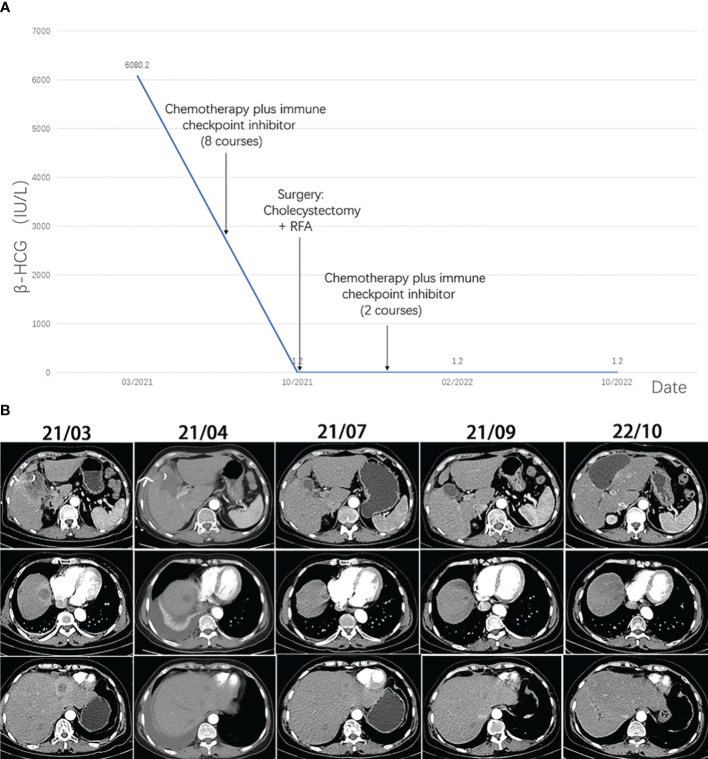
**(A)** The level of β-HCG during the treatment. **(B)** Computed tomography images of the lesions during the treatment.

**Figure 2:**
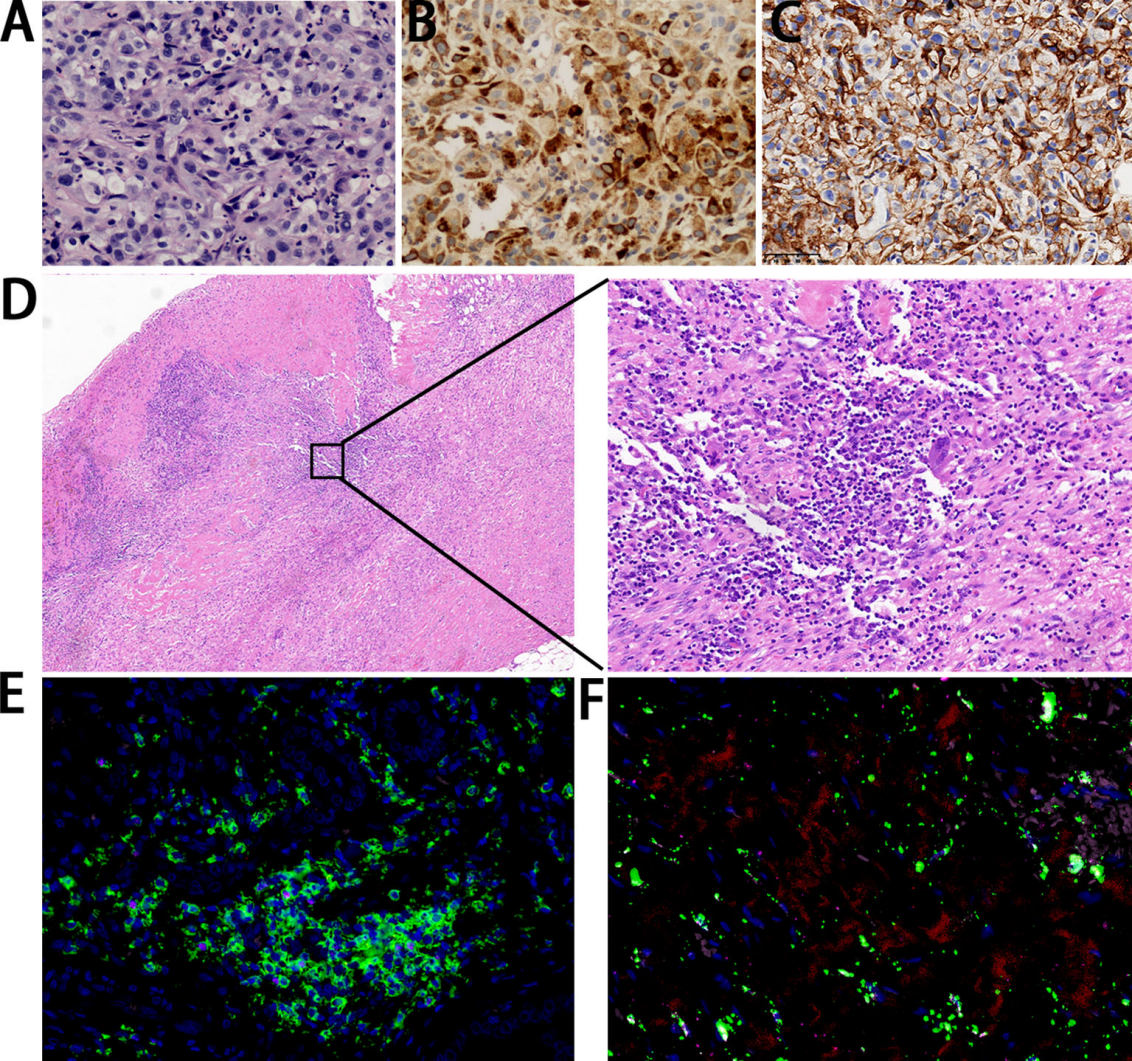
Pathological findings. **(A)** The metastatic lesion shows poorly differentiated carcinoma with large-scale necrosis (H&E ×200). **(B)** Almost all cancer cells show a positivity for HCG (×200). **(C)** Cancer cells show a strong positivity correlation for PD-L1 (×400). **(D)** The resected gallbladder shows no tumor cells with infiltration of lymphocytes and plasma cells (left: ×40; right: ×200). **(E)** Immunofluorescence staining of CD4 (green) in combination with CD3 (red), CD20 (pink), and FOXP3 (rose red) (×40). **(F)** Immunofluorescence staining of CD68 (green) in combination with CD8 (red), CD163 (pink), and CD56 (rose red) (×40).

In March 2021, a 58-year-old woman was admitted to our hospital because of a one-month history of right upper quadrant pain accompanied by fever. The tumor markers examination showed a significant increase in serum β-HCG level of 6080.2 IU/L (**Figure 1A**) (normal range<5.0 IU/L). Some inflammatory parameters were also significantly elevated: white blood cell, 11.2 × 10^9^/L with neutrophils occupying 79.3%; C-reactive protein, 52.3 mg/L (normal range<3 mg/L). CT of the abdomen revealed gallbladder cancer with invasion of the adjacent liver and multiple metastases in the liver (**Figure 1B**). No tumorous lesion, suggestive of a primary site, was identified in organs of the reproductive system by radiography (**Supplementary Figure 1**). A clinical diagnosis of cholecystitis and possible gallbladder tumor with liver metastases was made. Percutaneous cholecystostomy and needle biopsy of the liver were performed under ultrasound (US) guidance. Histopathological analysis of liver metastases showed poorly differentiated carcinoma with large-scale necrosis (**Figure 2A**). Immunohistochemical detection of various markers was further performed on the tumoral tissue and revealed strong positivity for HCG in most tumor cells (**Figure 2B**). Choriocarcinoma is usually composed of intermediate trophoblast cells, cytotrophoblast cells, and syncytiotrophoblast cells, and the interstitium is often accompanied by significant bleeding. The cell morphology of this case was uniform and did not have the characteristics of choriocarcinoma. Clinical examination of the female reproductive system showed no abnormality. After the additional examinations, the definite diagnosis of GBC with ectopic HCG with liver metastases was made.

After abdominal pain and fever improved, the patient received chemotherapy (gemcitabine 1.5g on days 1 and 8 and capecitabine 1g/1.5g on days 1-14, every 3 weeks) plus ICIs (carrellizumab 200mg on day 1) based on the guideline for the treatment of gallbladder carcinoma. The patient received a total of eight cycles of chemotherapy plus ICIs from April 2021 to September 2021. Contrast-enhanced CT was performed regularly, and the results suggested that the size of metastases and the extent of invasion were smaller than before (**Figure 1B**). The patient showed a good response to the treatment with mild side effects.

After eight courses of treatment, CT revealed a drastic reduction in the tumor size and the serum β-HCG level decreased to the normal range. Subsequently, radical resection of gallbladder carcinoma and radiofrequency ablation of liver lesions were performed. Histopathological analysis showed no tumor cells, indicating pathological complete response (PCR) was achieved. The infiltration of large amounts of lymphocytes and plasma cells was observed in the specimen (**Figure 2D**). Immunofluorescence staining showed positivity for CD4, CD8, and CD68 (**Figures 2E, F**).

The patient received two more courses of postoperative chemotherapy plus ICIs. To date, there was no evidence of tumor recurrence (**Figure 1B**) and the serum β-HCG level remained in the normal range (**Figure 1A**).

In order to investigate the genetic features of GBC with HCG production, gene analysis was further performed. Immunostaining showed tumor cells were positive for PD-L1 (Tumor cell Proportion Score, TPS ≥ 50%) (**Figure 2C**). WES of tumor tissue revealed a tumor mutation burden (TMB) of 1.51 mut/Mb, which was at a TMB-low level, and a copy number variation of 5.58%. Based on sequencing data, the tumor tissue was microsatellite stable (MSS). A total of eight mutations in the genes that drive tumors were also identified, namely, AXL 19q13.2, BARD1 p.P89fs, CCND3 6p21.1, NRAS p.Q61K, APC p.R805Q, JAK2 p.V392M, MCL1 1q21.3, and SMO p.R485Q.

**Figure 3:**
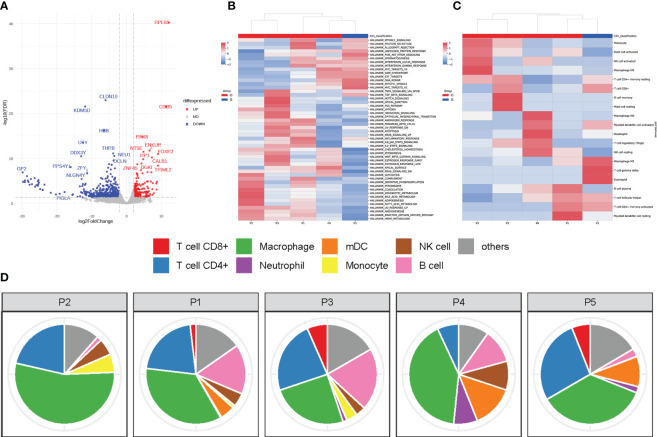
Immune microenvironment of GBC producing HCG. **(A)** Volcanic map for differentially expressed genes. (Red dots: significantly upregulated genes. Blue dots: significantly downregulated genes. Grey dots: non-differentially expressed genes.) **(B)** Heatmap showing the relative expression levels of genes in GBC with ectopic HCG and gallbladder adenocarcinoma without HCG. **(C)** Heatmap showing the relative abundances of infiltrating immune cells in GBC with ectopic HCG and gallbladder adenocarcinoma without HCG. **(D)** The proportions of infiltrating immune cells in GBC with ectopic HCG and gallbladder adenocarcinoma without HCG. (P5: GBC with ectopic HCG, P1-P4: gallbladder adenocarcinoma without HCG).

To better explore the differences in TIME features between GBC producing HCG and those without HCG, RNA-seq was performed, which refers to the use of high-through sequencing for cDNA sequencing to comprehensively obtain almost all transcripts of tissues of a tumor. The scatter plot was shown to demonstrate the variation of gene expression between two types of tumors (**Figure 3A**). A total of 734 differentially expressed genes were screened, which met the criteria of adjusted P ≤ 0.05 and fold changes of less than -2 or greater than 2. RPE65 and CLDN10 were, respectively, the most significantly upregulated protein and downregulated proteins.

The ssGSEA method was used to uncover the gene sets associated with the pathogenesis of GBC producing HCG. As shown in **Figure 3B**, MYC targets v1, mitotic spindle, DNA repair, PI3K/AKT/mTOR signaling, and unfolded protein response of gene sets were expressed in relatively higher levels in the present case than gallbladder adenocarcinoma without HCG. The immune infiltration features in our case and other cases of gallbladder adenocarcinoma were also analyzed using the CIBERSORT method, an analytical method to accurately estimate the immune composition of tumor tissue by using gene expression data. GBC producing HCG showed higher levels of infiltrating T cell gamma delta (4.5%), eosinophil (4.2%), and M1 macrophage (14.3%) than common gallbladder adenocarcinoma without HCG (**Figure 3C**). The dominant immune cell types in our case were macrophage and CD4+ T cells (**Figure 3D**).

## Discussions

GBC producing HCG is a rare and aggressive biliary malignancy with unsatisfactory treatment and poor prognosis. Despite a few cases that have been reported previously, our case is unique because (1): Primary GBC with ectopic HCG is exceedingly rare in the literature and may be more aggressive and more susceptible to metastasis than gallbladder adenocarcinoma (2). Due to its rarity, a standard treatment has not yet been established. Meantime, its aggressive nature and insensitivity to chemotherapy lead to its poor prognosis (3). In our case, the patient with advanced GBC producing HCG was treated with GC-based chemotherapy plus ICIs in which a PCR and the 1-year disease-free survival were obtained. To the best of our knowledge, this is the first case in which chemotherapy plus ICIs achieved a PCR in a patient with advanced GBC with HCG.

The majority of HCG-related carcinomas occur in the uterus and are related to a normal or abnormal pregnancy (1). They are also reported in extragonadal sites such as the gastrointestinal tract, lung, prostate, and breast (2–5). Primary GBC with HCG is extremely rare. Due to the rarity of this tumor, there are only a few case reports in the literature until now (6–13). Therefore, the clinical findings, pathological features, and optimal treatment are not well established. As Sato S et al. (11) reported, GBC with high HCG predominantly affects elderly women, with a mean age of 62.1 years, which is similar to patients with gallbladder adenocarcinoma (15). In addition, the clinical manifestations of GBC with HCG also resemble those of gallbladder adenocarcinoma, such as abdominal pain, fever, jaundice, and nausea (6–13). However, it can also present with specific symptoms, such as vaginal bleeding (10). Compared to adenocarcinoma, GBC with ectopic HCG is more aggressive, rapidly growing, and widely metastasizing. As highlighted by Li D et al. (16), the aggressive nature of the tumor is probably associated with HCG expression, which can significantly inhibit apoptosis and increase vascularization, causing the tumor cells to metastasize through the lymphatic and hematogenous routes.

Diagnosis of GBC with high HCG is difficult due to its unspecific symptoms and rare incidence. Studies show that the diagnosis is established in most patients after cholecystectomy (8, 9, 11). Because of the coexistence of adenocarcinoma and choriocarcinoma components, the value of a small specimen of percutaneous biopsy as a diagnosis tool for GBC with HCG is limited (12). Pathologically, most of the reported cases of GBC with HCG showed a coexistence of choriocarcinoma and adenocarcinoma components or cells of poorly differentiated adenocarcinoma expressing HCG (6–13). There are several theories regarding the origin of these choriocarcinomatous elements, which are still controversial. They may develop from displaced germ cells or totipotent rests, teratoma, or metastasis from the intra-uterine lesion (11). Another point of view states that they may develop from retro-differentiation of gallbladder adenocarcinoma, which is the widely accepted hypothesis (7).

To date, there is no consensus regarding the optimal treatment for GBC with HCG due to its rarity. In most reported cases, surgery or chemotherapy alone is the most common treatment for this disease (6–13). However, the cases responded unsatisfactorily to the aforementioned treatment strategy, showing a short survival. Chemotherapy regimens, used successfully for gestational choriocarcinoma, appear to be less effective in the treatment of non-gestational choriocarcinoma (6–8, 14). However, Wang JC et al. (9) found that cisplatin-based chemotherapy might be effective for non-gestational choriocarcinoma, which prolongs the survival of patients. Sato S et al. (11) reported a case treated with surgery plus Gemcitabine-based chemotherapy that achieved remission and had more than 11 months of progression-free-survival. Because only a few cases have been reported in the literature until now, the optimal treatment is not yet well established and further research is needed.

In our case, the patient was treated with GC-based chemotherapy, the first-line treatment for advanced GBC, combined with ICIs (17). To explore the underlying reasons for the durable complete response in this case, we performed a genetic analysis of tumor tissue from the patient to understand the tumor immune-microenvironment. Rizzo A et al. (18) summarized that PD-L1, TMB, and microsatellite instability (MSI) are emerging biomarkers predictive of the response to ICIs. Patients with PD-L1 expression and MSI-H tend to respond well to ICIs, leading to better prognosis (18, 19). In the present case, high PD-L1 expression in tumor tissue could be responsible for the good response to ICIs. The TIME is another biomarker that predicts the response to ICIs (18).

Tumor-associated macrophages are the main tumor-invasive immune cell population in TIME. Among them, M2 macrophages promote tumor growth and metastasis, which are associated with poor prognosis. On the contrary, M1 macrophages have the ability to act as anti-tumor agents (20). Zeng D et al. found that the high infiltration of M1 macrophages is significantly correlated with a better response to ICIs and prolonged survival (21). Compared to other biliary adenocarcinoma cases, the proportion of M1 macrophages is significantly higher in our case, which may explain the good prognosis of our case. In addition, some studies reported that higher T cell infiltration occurs together with increased PD-1 and PD-L1 expression, which indicates a good response to ICIs (22, 23). Therefore, the relatively high proportion of T cells in our case may be one of the explanations for the good therapeutic response. Furthermore, immunofluorescence staining of resected gallbladder showed positivity for CD4, CD8, and CD68, but less positivity for M2 macrophage marker CD163. The results indicated the infiltration of CD8+ T cell, CD4+ T cell, and M1 macrophages, consistent with the features of immune infiltration analyzed by the CIBERSORT method.

In addition, the mechanisms regulated by several oncogenic signaling pathways can contribute to the overexpression of PD-L1 in cancers. The PI3K–AKT–mTOR pathway is a central regulator of mRNA translation and mTOR can upregulate the expression of PD-L1 (24). In addition, MYC also upregulates PD-L1 at the transcriptional level. Overexpression of MYC evades upstream open reading frames (uORF)-mediated repression of PD-L1 translation by enhancing the eukaryotic translation initiation factor 2 complex-α subunit (eIF2α) phosphorylation, thus leading to increased PD-L1 expression (25). As shown in **Figure 3C**, both of these oncogenic signaling were highly expressed in our case, which may explain the good response to ICIs.

In conclusion, we report a case of advanced GBC producing HCG in which chemotherapy plus ICIs achieved a PCR. Furthermore, this is also the first report that described the tumor genetic feature and TIME atlas of HCG-producing GBC. Although this single-case observation does not allow for a firm conclusion on this disease, it can provide the possibility of chemotherapy plus ICIs as a potentially curative option. The clinical effects of such treatment in GBC with HCG, as well as the TIME feature, deserve further investigation.

## Data availability statement

The datasets presented in this study can be found in public repositories. The whole sequence data reported in this paper available in the National Center for Sequence Read Archive, Biotechnology Information, under accession number SRR26047214 at https://www.ncbi.nlm.nih.gov/sra.

## Ethics statement

The studies involving humans were approved by Human Research Ethics Committee of Fourth Affiliated Hospital of Zhejiang University. The studies were conducted in accordance with the local legislation and institutional requirements. The participants provided their written informed consent to participate in this study. Written informed consent was obtained from the individual(s) for the publication of any potentially identifiable images or data included in this article.

## Author contributions

YBL, XD, and BZ guided and supervised the project. QW and YL accomplished the collection and analysis of clinical data. SJ and SW accomplished the collection and analysis of pathology data. YBL and YM managed the patient. QW and BZ wrote and revised the manuscript. All authors contributed to the article and approved the submitted version.

## Supplementary material

Supplementary Figure 1
**(A)** Enhanced Computed tomography images of the reproductive system at the time of diagnosis. **(B)** Enhanced Magnetic resonance imaging of the reproductive system at the time of diagnosis (coronal plane). **(C)** Enhanced Magnetic resonance imaging of the reproductive system at the time of diagnosis (sagittal plane).

Supplementary Figure 2 Comparison of enhanced computed tomography images before and after combined immunotherapy (Red row: abdominal metastases).

Supplementary Table 1 Summarization of pathological findings of our case. TMB: tumor mutational burden; MSS: microsatellite stable; CNV: copy number variation
