# Supplementary material for: Case Report: Complete pathological remission of human chorionic gonadotrophin-producing gallbladder carcinoma with multiple liver metastases after treatment with chemotherapy plus an immune checkpoint inhibitor
Source: Front Immunol. 2023 Sep 29;14:1173520. doi: 10.3389/fimmu.2023.1173520 (PMC10570427; doi:10.3389/fimmu.2023.1173520)
Supplement: Supplementary Figure 1 — (A) Enhanced Computed tomography images of the reproductive system at the time of diagnosis. (B) Enhanced Magnetic resonance imaging of the reproductive system at the time of diagnosis (coronal plane). (C) Enhanced Magnetic resonance imaging of the reproductive system at the time of diagnosis (sagittal plane). [file DataSheet_1.zip › supplemental materials/Suppl Table 1.pdf]

Suppl. Table 1: Summarization of pathological findings of our case.

	liver metastases<br><br>(pre-treatment)		Gallbladder<br><br>(post-treatment)
Pathology	poorly differentiated carcinoma with necrosis;	Pathology	No tumor cells;<br><br>Infiltration of many lymphocytes and plasma cells;
Immuno-staining	HCG (+);<br><br>PD-L1 (+)	Immunofluorescence staining	CD4 (+), CD8 (+), CD68 (+)
Whole-exome sequencing	TMB (1.51mut/Mb)<br><br>MSS<br><br>CNV (5.58%)<br><br>8 mutations in driving-tumor genes		

TMB: tumor mutational burden; MSS: microsatellite stable; CNV: copy number variation
