# Supplementary figures and images for: Case Report: Complete pathological remission of human chorionic gonadotrophin-producing gallbladder carcinoma with multiple liver metastases after treatment with chemotherapy plus an immune checkpoint inhibitor
Source: Front Immunol. 2023 Sep 29;14:1173520. doi: 10.3389/fimmu.2023.1173520 (PMC10570427; doi:10.3389/fimmu.2023.1173520)

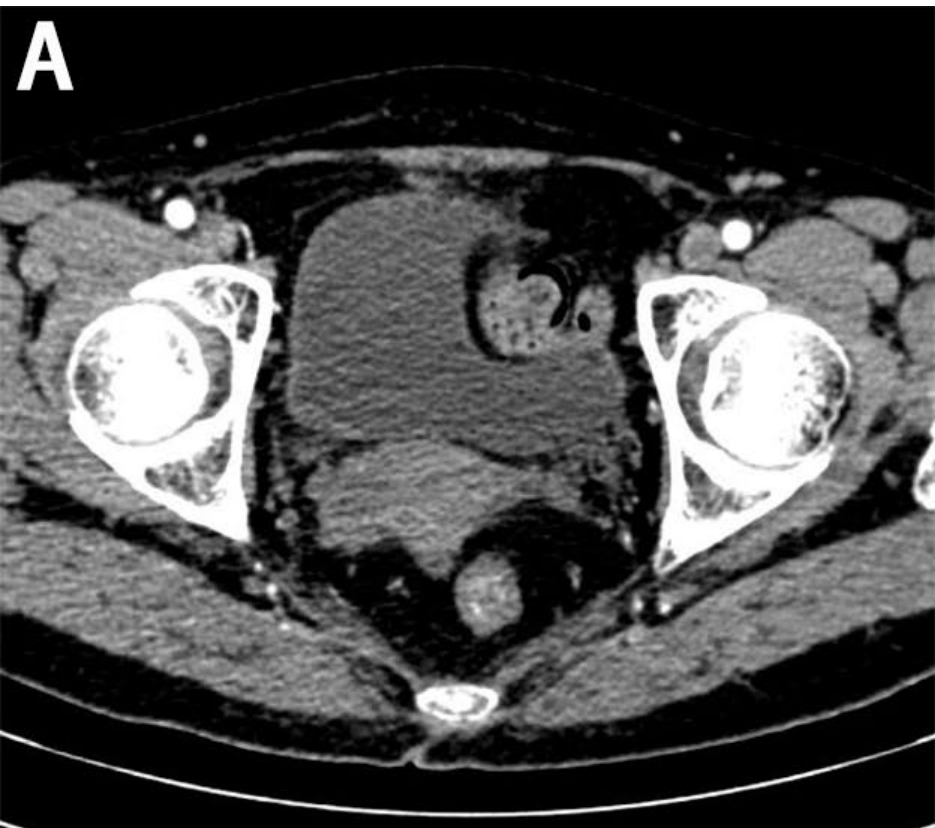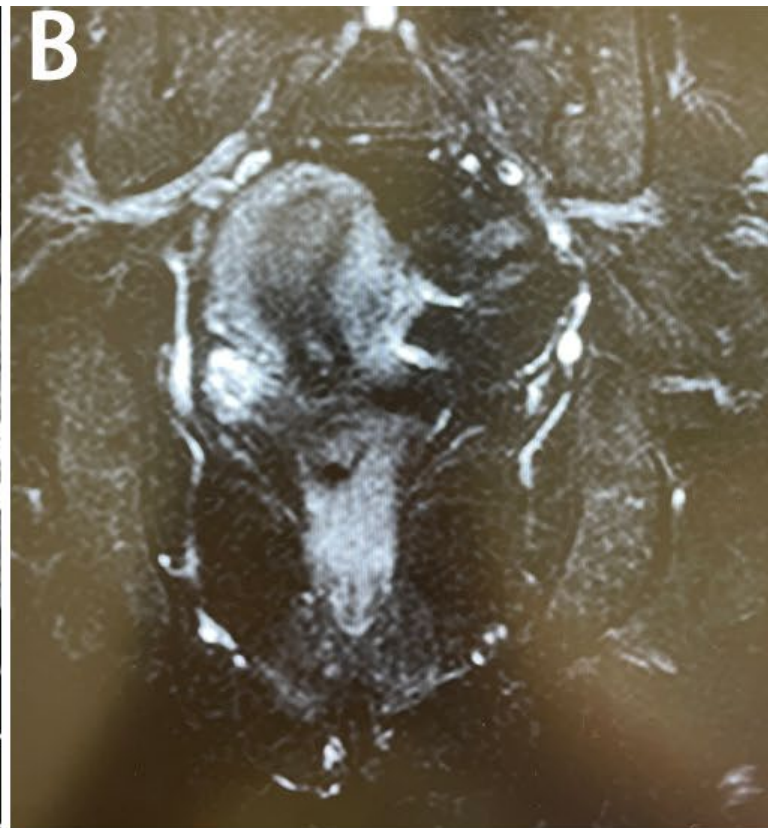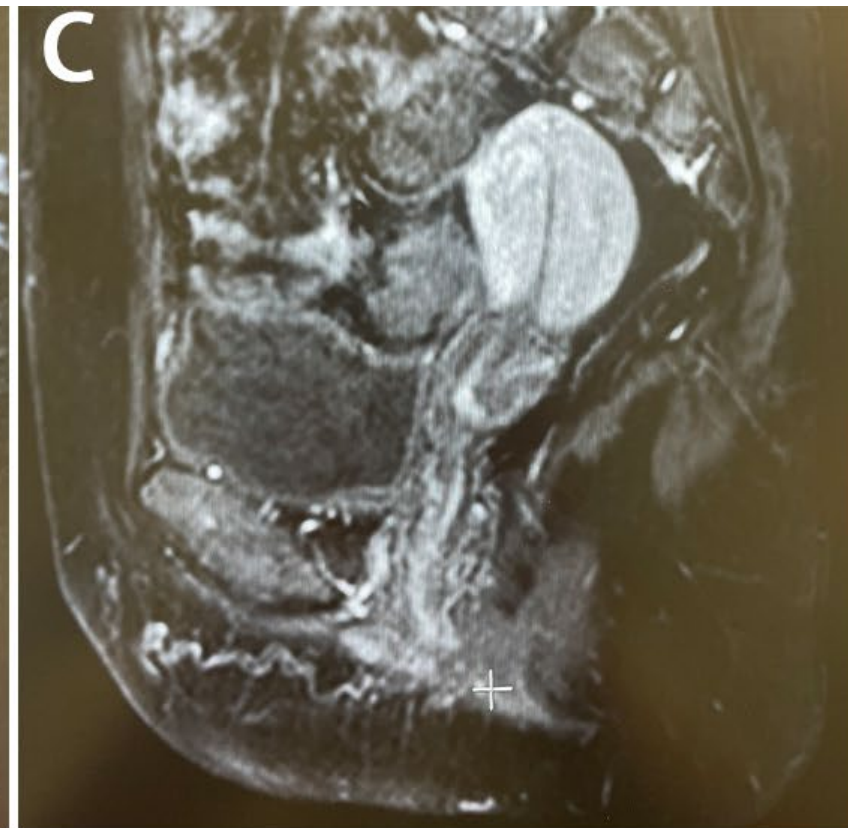

Supplement: Supplementary Figure 1 — (A) Enhanced Computed tomography images of the reproductive system at the time of diagnosis. (B) Enhanced Magnetic resonance imaging of the reproductive system at the time of diagnosis (coronal plane). (C) Enhanced Magnetic resonance imaging of the reproductive system at the time of diagnosis (sagittal plane). [file DataSheet_1.zip › supplemental materials/Suppl. Figure 1.pdf]

21/03

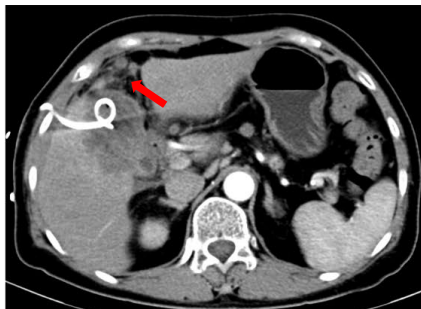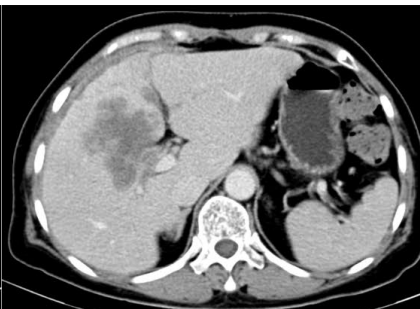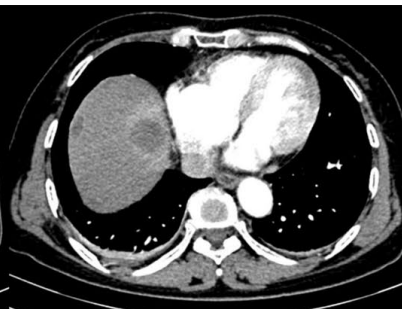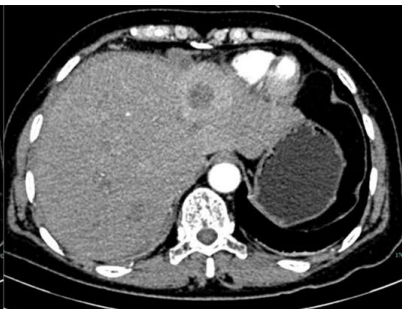

21/09

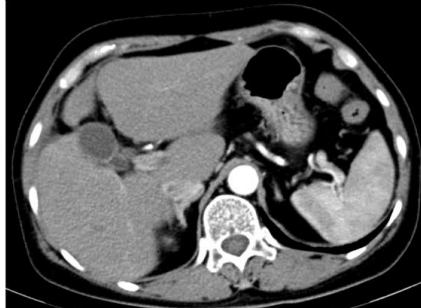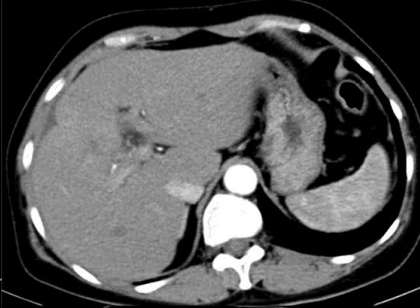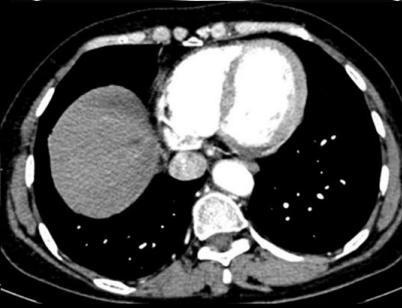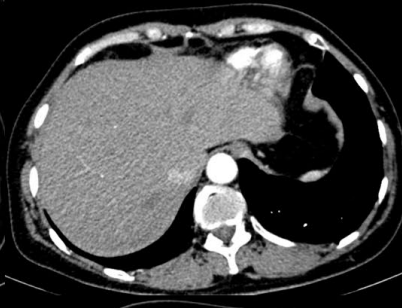

22/10

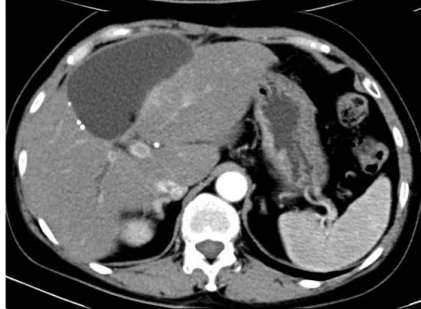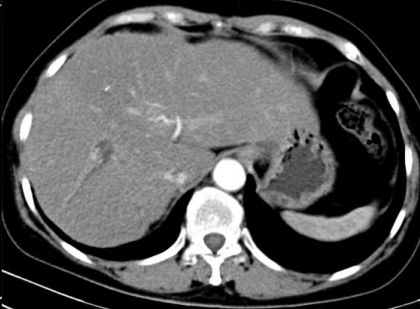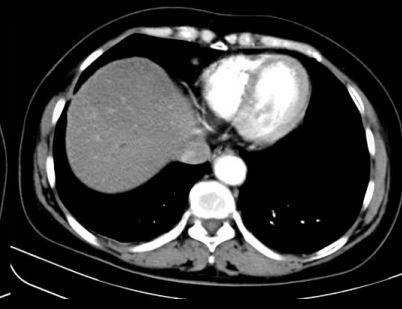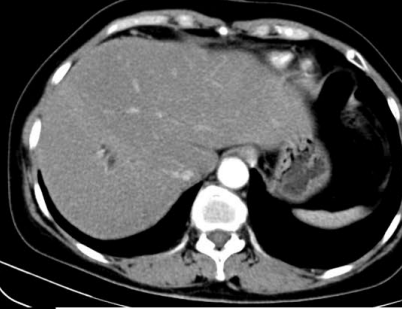

21/03

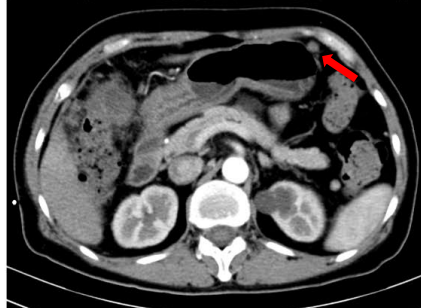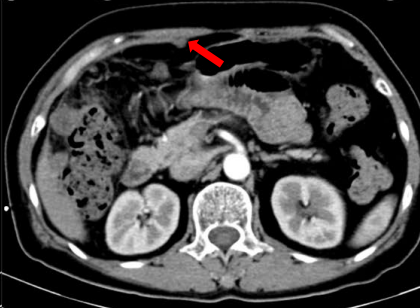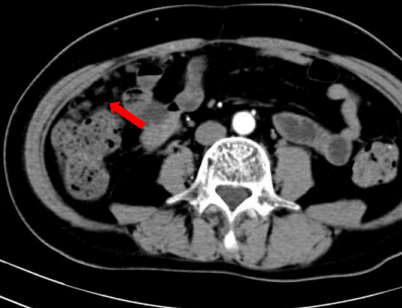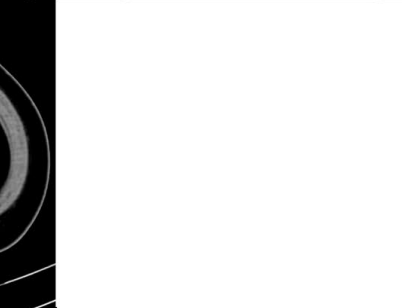

21/09

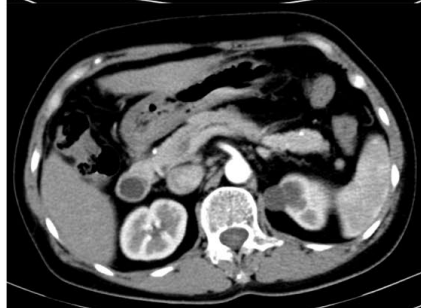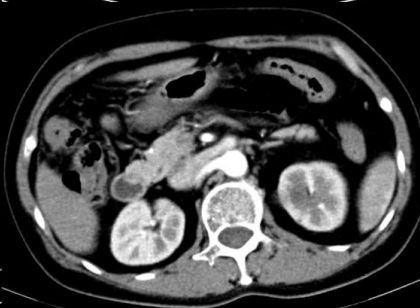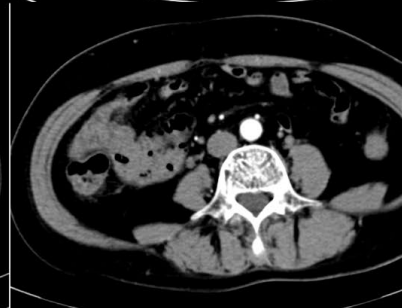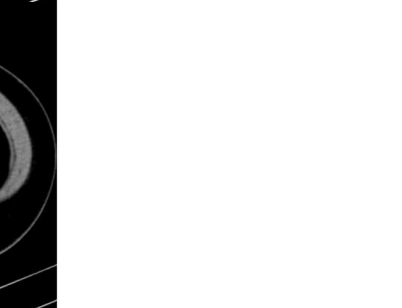

22/10

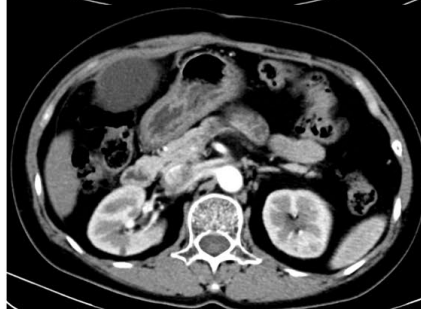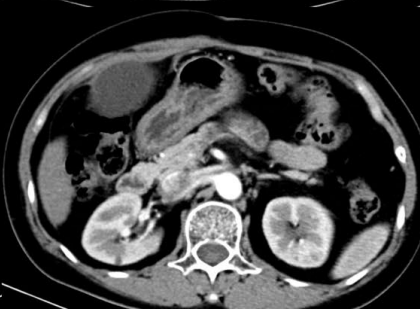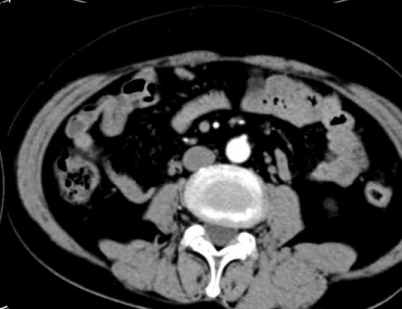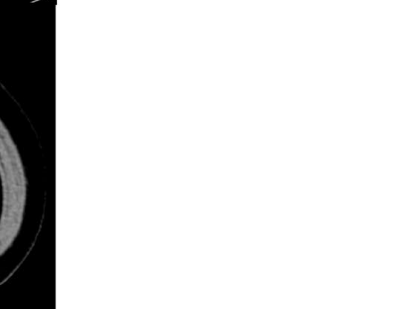

Supplement: Supplementary Figure 1 — (A) Enhanced Computed tomography images of the reproductive system at the time of diagnosis. (B) Enhanced Magnetic resonance imaging of the reproductive system at the time of diagnosis (coronal plane). (C) Enhanced Magnetic resonance imaging of the reproductive system at the time of diagnosis (sagittal plane). [file DataSheet_1.zip › supplemental materials/Suppl. Figure 2.pdf]
